# Microencapsulation and Bioaccessibility of Phenolic Compounds of *Vaccinium* Leaf Extracts

**DOI:** 10.3390/antiox11040674

**Published:** 2022-03-30

**Authors:** Bianca Eugenia Ștefănescu, Silvia-Amalia Nemes, Bernadette-Emőke Teleky, Lavinia Florina Călinoiu, Laura Mitrea, Gheorghe Adrian Martău, Katalin Szabo, Mihaela Mihai, Dan Cristian Vodnar, Gianina Crișan

**Affiliations:** 1 Department of Pharmaceutical Botany, “Iuliu Hațieganu” University of Medicine and Pharmacy, 400337 Cluj-Napoca, Romania; stefanescu.bianca@umfcluj.ro (B.E.Ș.); gcrisan@umfcluj.ro (G.C.); 2 Life Science Institute, University of Agricultural Sciences and Veterinary Medicine Cluj-Napoca, 400372 Cluj-Napoca, Romania; bernadette.teleky@usamvcluj.ro; 3 Institute of Life Sciences, Faculty of Food Science and Technology, University of Agricultural Sciences and Veterinary Medicine, 400372 Cluj-Napoca, Romania; amalia.nemes@usamvcluj.ro (S.-A.N.); lavinia.calinoiu@usamvcluj.ro (L.F.C.); laura.mitrea@usamvcluj.ro (L.M.); adrian.martau@usamvcluj.ro (G.A.M.); katalin.szabo@usamvcluj.ro (K.S.)

**Keywords:** *Vaccinium*, microencapsulation, chlorogenic acid, maltodextrin, viscosity, in vitro digestion

## Abstract

In recent years, *Vaccinium* spp. (bilberry-VMT, lingonberry-VVIT, and blueberry-VCS) have sparked particular interest for their prospective health benefits. The latest investigations have place them as important alternative sources of nutraceuticals as their leaves are the main by-products of berry harvesting. The present study is aimed at investigating the bioaccessibility of phenolic compounds from leaves of the *Vaccinium* species, both as microencapsulated powder and aqueous extracts, following exposure to in vitro simulated digestion. Moreover, the impact of maltodextrin and glucose microencapsulation carriers on the extracts’ phenolic content was assessed. Prior to encapsulation, the viscosity of the emulsions was shown at a shear stress of 50 s^−1^ dilatant and a Newtonian behaviour above this value with a final viscosity between 1.024 and 1.049 mPa·s. The final microencapsulation yield for the samples ranged between 79 and 81%. Although the microencapsulated forms presented a targeted release at the intestinal level, the phenolic content decreased after gastrointestinal digestion. The bioaccessibility of the microencapsulated extracts showed higher values than their non-encapsulated counterparts, with the highest value of 45.43% in the VVIT sample, followed by VCS with 41.07%. However, the non-encapsulated VCS sample presented high bioaccessibility after in vitro digestion (38.65%). As concluded, further in vivo research should be conducted on the leaves of the *Vaccinium* species.

## 1. Introduction

Production and demand for supplements containing plant-based bioactive chemicals are steadily increasing, as revealed by several scientific studies that confirm a link between the consumption of products rich in bioactive compounds and the prevention of certain chronic diseases [1,2,3,4].

The fruits of the *Vaccinium* genus have long been among the most studied berries, due to their varied phenolic compounds [5,6,7]. Moreover, *Vaccinium* leaves are the principal by-product of berries and contain more phenolics than any other tissue of the species, including the fruit [8,9,10]. The *Vaccinium* genus belongs to the Ericaceae family [11] and it consists of wild species, such as bilberry-*Vaccinium myrtillus* L., lingonberry-*Vaccinium vitis-idaea* L., and culture species, such as blueberry-*Vaccinium corymbosum* L. [12].

Bilberry, also called European blueberry or whortleberry, is a small perennial shrub native to Europe [12,13,14]. Bilberry leaves have commonly been used for their antidiabetic properties and as a remedy for urinary tract affections [15]. Moreover, recent in vivo studies have proven the hypoglycaemic and hypolipidemic activities of the bilberry leaf [16,17]. Bilberry leaves are also a significant source of polyphenols, including hydroxycinnamic acids, catechins, flavonols, proanthocyanidins, occasional anthocyanins, cinchonains, and iridoids [5,18,19]. Lingonberry, also called cowberry or partridgeberry, is a small green shrub that grows throughout the year. Like bilberry leaves, lingonberry leaves are used to prevent and treat urinary tract affections due to their high phenolic content [20]. Anti-cough and phlegm-removing effects [21,22], neuroprotective activity [23], antioxidant, antibacterial, and antimutagenic activities are some of the other biological properties of lingonberry leaves [6,19,24]. As such, the phenolic content of lingonberry leaves is comparable to that observed in bilberry leaves. Additionally, flavonols, phenolic acids, arbutin derivatives, proanthocyanidins, catechins, iridoids, and cinchonains are amongst the phenolic compounds found in its chemical composition [6,20,25].

*Vaccinium corymbosum* L., often known as northern highbush blueberry, is a worldwide cultivated shrub. Its leaves have been granted less attention than bilberry and lingonberry leaves, although literature research has revealed that blueberry leaves are high in phenolic compounds, as well [25,26,27,28]. This was proven by a recent study from the same research group, who identified four types of phenolic chemicals in blueberry leaves: flavonoids, flavonols, hydroxycinnamic acids, and anthocyanins [19].

*Vaccinium* leaf extracts can become prospective ingredients and additives for different industries, especially in plant-based supplements or products, due to their varied phenolic content. However, phenolic chemicals are sensitive and may alter in response to environmental or gastrointestinal factors [29]. In this respect, microencapsulation is a well-known technique used to preserve phenolic compounds by entrapping them in a matrix that ensures a targeted release during gastrointestinal digestion [30]. Microencapsulation is a mechanical and physicochemical process where a substance is entrapped into a coating material to produce particles of different sizes, from a few nanometres to a few millimetres [31,32,33]. The encapsulated compound, known as core material, is distributed in a matrix known as coating or shell. Thus, microencapsulating phenolic-rich extracts aims to protect the bioactive compounds, to achieve gastrointestinal tract absorption and obtain the purposed effect [30]. In the case of phenolic-rich extracts, the carrier material should be food grade and capable of creating a solid barrier for cell protection; therefore, gum Arabic and starches are the most commonly used matrix [29].

In this respect, the spray-drying technique is the most common and least expensive microencapsulation method used for thermally sensitive foods and pharmaceutical products [3,13]. During spray drying, a dry powder is produced by quickly spraying a slurry liquid using hot gas. The primary key involving the spray drying process is the high temperature used, while exposure time is low (few seconds) with maltodextrin playing a protective role. Even so, a part of the phenolic compounds might be affected. Numerous improvements have been made to the process, including spray freeze drying, spray chilling, ultrasonic vacuum spray drying, and others [34,35,36,37].

In this present context, where diet-related illnesses have become more common in recent decades, various clinical investigations have been conducted to link patients’ diet to their clinical condition [38,39]. Understanding the physiological reaction to particular compounds requires a comprehensive insight into the various digestive processes in the human gastrointestinal system. This can be achieved by invasive procedures such as aspiration from the stomach or small intestine and through more minor invasive procedures such as imaging methods (e.g., magnetic resonance imaging) [40]. In vivo clinical studies involving human or animal models may be difficult to perform, as well as unsuitable and costly, or lacking ethical grounds. Therefore, in vitro models have been used to simulate digestion. Static, semi-dynamic, and dynamic models are the three types of in vitro digesting techniques that are frequently utilized. Static models, which employ a fixed ratio of extracts to enzymes and electrolytes, as well as a constant pH for each digestion phase, have been widely used with food and drug applications for decades due to their simple design [41].

The main objective of this research was to employ the spray-drying microencapsulation method, using maltodextrin and glucose as coating materials to preserve phenolic- *Vaccinium* leaf extracts. In addition, the microencapsulation impact on the bioaccessibility of phenolic chemicals was examined using in vitro simulated digestion.

## 2. Materials and Methods

### 2.1. Plant Material

The leaves of the three *Vaccinium* species (*V. myrtillus* L., *V. corymbosum* L. and *V. vitis-idaea* L.) were collected from northern Romania in the autumn of 2017, dried at room temperature for 7–10 days, and stored in the dark, prior to their analysis. Bilberry (*Vaccinium myrtillus* L.) and lingonberry (*Vaccinium vitis-idaea* L.) leaves were collected from spontaneous flora, randomly sampled from ca. 10 shrubs in the same 20 m × 20 m area. These plant species were identified by Gianina Crisan, Professor in Botanical Sciences (Pharmaceutical Botany), while the numbers of Plant Voucher Specimens are VM103 and VVI105.

The *V. corymbosum* L. leaves are from a cultivar variety (controlled drip irrigation system, acid brown soil).

### 2.2. Chemicals

Catechin, chlorogenic acid, quercetin, and cyanidin chloride were acquired from Sigma-Aldrich as standard chemicals for HPLC-DAD-ESI-MS examination (Steinheim, Germany). Acetic acid, acetonitrile, and maltodextrin were also acquired from the same supplier. VWR Chemicals in Dresden, Germany, supplied the D-glucose. The materials used in the spray-drying process were of analytical grade: maltodextrin (Sigma-Aldrich, Steinheim, Germany) and D-glucose (VWR Chemicals, Dresden, Germany). The reagents used for in vitro digestion, as well as pepsin from porcine gastric mucosa (Art. No. P6887), pancreatin from porcine pancreas (Art. No. P7545), and bovine bile extract (Art. No. B8631) were purchased from Sigma-Aldrich (Taufkirchen, Germany); additional chemicals needed for the preparation of simulated salivary, gastric and intestinal fluids were of analytical grade. Double distilled water was used to prepare all solutions and emulsions.

### 2.3. Aqueous Extraction

*Vaccinium* plant leaves, abbreviated as VMT (*V. myrtillus* L.), VCS (*V. corymbosum* L.), and VVIT (*V. vitis-idaea* L.), were finely powdered using an automatic grinder (Caso, Arnsberg, Germany) and 10 g of each sample were homogenized with 100 mL of distilled water. The bioactive compounds were extracted from the *Vaccinium* plants through ultrasonication (Elmasonic E15H, Elma, Singen, Germany) for 1 h at 80 °C. The samples were then centrifuged (Eppendorf 5810 R, Eppendorf AG, Hamburg, Germany) twice at 11,000 rpm for 10 min to advance the extraction of the bioactive compounds. The separated supernatant was collected and stored at −20 °C until further analysis [42,43].

### 2.4. HPLC-DAD-ESI-MS Identification and Quantification

All leaf extracts from the selected *Vaccinium* species were subjected to HPLC-DAD-ESI-MS analysis for phenolic compound identification and quantification [44,45]. The system consisted of an Agilent 1200 HPLC with DAD detector and an MS-detector single-quadrupole Agilent 6110 was used. The following XDB C18 Eclipse column (4.6 × 150 mm, particle size 5 μm) (Agilent Technologies, USA) was selected for phenolic fragmentation at room temperature. Two gradients composed of 0.1% acetic acid in distilled water (*v*/*v*) (solvent A) and 0.1% acetic acid in acetonitrile (*v*/*v*) (solvent B) were used at a flow rate of 0.5 mL/min, following the elution program detailed by Dulf et al. [46]. A scanning range of 100–1200 *m*/*z* in the ESI (+) mode was applied for MS fragmentation, setting the capillary voltage at 3000 V, 350 °C, and the nitrogen flow at 8 L/min. Additionally, DAD was used to monitor the eluent, while the absorbance spectra (200–600 nm) were registered during each run. The Agilent ChemStation Software (Rev B.04.02 SP1, Palo Alto, CA, USA) was used to analyse the data. A comparison between the retention times, UV visible, and mass spectra of the peaks with 4 reference standards was performed for accurate and correct identification of each phenolic compound, as follows: the anthocyanin compounds were quantified using the calibration curve performed with cyanidin on concentration ranges between 10–100 μg/mL and expressed as cyanidin/g equivalents (mg cyanidin/g plant material) (r^2^ = 0.9951); the compounds belonging to the hydroxycinnamic acid subclass were quantified using the calibration curve performed with chlorogenic acid on the concentration range of 10–50 μg/mL, expressed as chlorogenic equivalents (mg chlorogenic acid/g plant material) (r^2^ = 0.9937); the compounds from the flavonol group were quantified using the calibration curve performed with quercetin on the 10–200 μg/mL concentration ranges expressed as quercetin equivalents (mg quercetin/g plant material) (r^2^ = 0.9951), while the compounds of the flavanol subclass were quantified using the calibration curve performed with catechin standard on the 10–200 μg/mL concentration ranges and expressed as catechin equivalents (mg catechin/g plant material) (r^2^ = 0.9985).

### 2.5. Rheological Measurements

To examine the influence of each extract prior to microencapsulation, the viscosity of the aqueous solutions was measured. Both the viscosity and flow behaviour were measured through a modular compact Anton Paar MCR 72 rheometer (Anton Paar, Graz, Austria) [47,48,49], equipped with a concentric cylinder system (C–PTD 150/XL/AIR/18P) and a double-gap measuring system (temperature range from 5 °C to 150 °C). For each sample solution, 15 mL were poured into the double-gap system of the rheometer. Each measurement was operated at 25 °C, with an increasing shear rate from 5 to 300 s^−1^ (logarithmic ramp) and a determination value from 0 to 100 s^−1^ (logarithmic ramp). Subsequently, the RheoCompassTM software was applied in order to investigate the results obtained after the rheological analyses. The measurements were performed in triplicate and the results are indicated as mean ± SD [50,51].

### 2.6. Microencapsulation Process (Spray-Drying, Atomization)

#### 2.6.1. Carrier Preparation

In order to obtain an utterly homogenized carrier before spray-dryer feeding, maltodextrin (80%), glucose (20%), and plant extracts (10%) were mixed on a magnetic plate at 60 °C for 30 min (250 rpm). In addition, to minimize viscosity and avoid nozzle blockage during atomization, the carriers were diluted twice with water and homogenized for 3 min before being transferred to the spray-dryer [37].

#### 2.6.2. Microencapsulation

The powder formulation was carried out using a ‘BUCHI Mini Spray Drayer B-290’ laboratory spray-dryer (BÜCHI Labortechnik AG, Flawil, Switzerland). The working parameters were as follows: solution volume flow of 5 mL/min, compressed air volume flow of 357–450 L/h, a suction flow rate of 35 m^3^/h, the atomization temperature of 140 °C, and the outlet air temperature was monitored at 90 ± 5 °C. The spray-dryer nozzle had a 0.7-mm-diameter and a 60 × 50 × 110 mm main spray chamber. The resulting plant-based microcapsules collected in the cyclone were recovered and stored in plastic jars, in dry conditions, at room temperature and protected from light until further analysis. The microencapsulation yield was determined using the following formula: *W_f_*/*W_i_* ×100, where *W_f_* represents the amount of recovered powder (final weight, in grams), and *W_i_* represents the amount of initial dried substance (initial weight, in grams) [33,52].

### 2.7. Bioaccessibility of Phenolic Compounds of Vaccinium spp. Leaves during Simulated Digestion

The research consisted of 6 samples (3 aqueous extracts, 3 microencapsulated extracts) obtained from each *Vaccinium* species and microcapsules of the extracts obtained after the spray drying process, which underwent the in vitro digestion model in order to investigate the phenolic compounds. The working protocol developed by INFOGEST 2.0 [53] involved three distinct stages: oral phase, gastric phase, and intestinal phase.

The oral phase of the simulated digestion, with a standardized 1:1 (*wt*/*wt*) ratio of sample to simulated oral fluid, was conducted without α-amylase, as the samples did not contain starch. Simulated salivary fluid (SSF) was composed of electrolyte stock solutions of KCl, KH_2_PO_4_, NaHCO_3_, MgCl_2_•6H_2_O, (NH_4_)_2_CO_3,_ and water, as previously described by Brodkorb et al. in the international consensus of standardized in vitro digestion method [53], with an added CaCl_2_(H_2_O)_2_ solution and pH adjustment to 7. The samples underwent oral phase for two minutes at 37 °C in a shaking incubator (New Brunswick Innova 44, Eppendorf AG, Hamburg, Germany) at 95 rpm.

During the gastric phase, the simulated gastric fluid (SGF) was pre-heated to 37 °C and added to the oral bolus in a ratio of 1:1 (vol/vol). The SGF was composed of electrolyte solutions KCl, KH_2_PO_4_, NaHCO_3_, MgCl_2_•6H_2_O, (NH_4_)_2_CO_3_, as described in the INFO-GEST protocol [53], alongside a CaCl_2_(H_2_O)_2_ solution (0.03 M), porcine pepsin solution (2000 U/mL in the final digestion mixture) and water. The pH of the samples was adjusted to 3 by adding HCl (1 M) and the mixture was homogenized and incubated for 2 h in a shaking incubator (95 rpm).

For the intestinal phase of the in vitro digestion, pre-warmed simulated intestinal fluid (SIF) was added to the gastric chime to achieve a final ratio of 1:1 (*v*/*v*). SIF was composed of electrolyte stock solutions as described in the INFOGEST protocol [53], with added bile extract solution, in order to reach a final concentration of 10 mM. The pH was set to 7 using NaOH (1 M), and the mixture was homogenized and incubated for 2 h at 37 °C in a stirring incubator set to 95 rpm.

Samples were collected from all three simulated digestion phases, immediately centrifuged for 60 min at 4800× *g* and 4 °C (Eppendorf 5810 R, Eppendorf AG, Hamburg, Germany) to minimize enzyme activity and to remove the undigested material. The supernatant, considered to contain the phenolic compounds released from the microcapsules, respectively, from the extracts, was membrane-filtered (0.2 μm Millipore nylon filter) to separate the micellar fraction and underwent qualitative and quantitative analysis of phenols by HPLC-DAD-ESI-MS, as described in Section 2.4. The phenolic values found were normalized for the dilution factor induced by the addition of digestive fluids.

### 2.8. Statistical Analysis

All of the analyses were performed in triplicate, and the data were reported as the means ± standard deviation (SD). The statistical differences between the aqueous leaf extract and the microencapsulated powder, individual for each type of the three species, before and after each digestion phase, were performed using one-way analysis of variance (ANOVA) (Tukey multiple comparison tests) via GraphPad Prism Version 8.0.1 (Graph Pad Software Inc., San Diego, CA, USA). Differences between means at the 5% level were reported to be statistically significant.

## 3. Results and Discussions

### 3.1. Rheological Measurements of Aqueous Extracts of Vaccinium spp. Mixed with Microencapsulation Carrier

**Figure 1:**
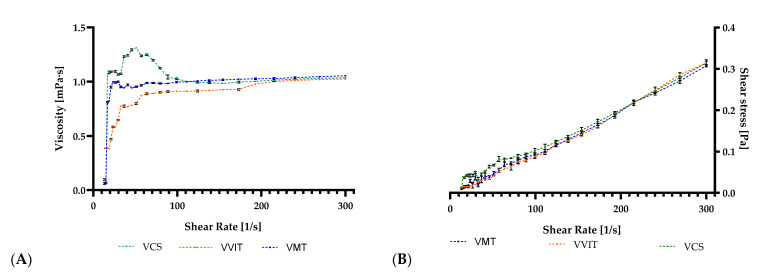
The (**A**) viscosity and (**B**) shear stress of aqueous solutions with the increase of shear rate, Abbreviations used: VMT (*V. myrtillus* L.), VCS (*V. corymbosum* L.), and VVIT (*V. vitis-idaea* L.)—plant species; Values (expressed as mean values ± SD, *n* = 3).

Following centrifugation, the aqueous extracts of *Vaccinium* spp. were recovered in volumes of 55 mL for the VMT sample, 64 mL for the VCS sample, and 50 mL for the VVIT sample. The consequence of concentration on the viscosity of these aqueous extracts was investigated between a shear rate of 0–300 s^−1^. The characteristics of these extracts alongside solvent nature and wall material can influence spray-drying properties. A low viscosity coupled with a high surface tension can result in appropriate microcapsules [54]. At a shear rate under 50 s^−1^, a shear-thickening (dilatant) behaviour was displayed, while above this value the emulsions began to display a Newtonian behaviour. It was regularly found that after reaching a particular shear rate, extract viscosity began to increase at lower solid concentrations [55]. Viscosity ranges for all examined products were 1.024–1.049 mPa s at a shear rate of around 300 s^−1^ (Figure 1). Similar results were observed through the microencapsulation of Vitamin A, with different wall materials (gelatine-sucrose and gelatine-peach-gum-sucrose), although these pastes did not reveal a Newtonian behaviour, but mostly a shear thinning behaviour [56]. There was no noticeable difference in viscosity across the aqueous solutions in the present study. Moreover, the degree of shear thickening affects the viscosity of the substance.

Emulsion viscosity is critical because it is proportional to particle diameter. To achieve smaller droplet sizes, the atomization energy delivered to the nozzle must be higher than the viscous forces present. As such, the result of the viscous forces’ tendency to reduce the energy available for breaking the droplets apart consists of bigger droplets. Maltodextrin’s low viscosity, even at high concentrations, has significant advantages, including high availability, low cost, neutral flavour, and excellent protection against aroma oxidation [57]. The same behaviour and low viscosity could be observed in similar studies where maltodextrin was used as an encapsulating agent [58,59]. As proven in the study by Gimbun et al., 2018, an increased viscosity produces a spray-dried powder with greater moisture content [58]. The viscosity of tamarillo juice emulsions was also low with maltodextrin as carrier material having 9.65 ± 0.12 MPa·s [60]. In contrast, the spray-drying of carrot juice with gum Arabic maltodextrin and whey protein isolates in different mixtures all produced sheer-thinning emulsions, and greater viscosities than is common for the particular fluid type, thus having greater dry matter content [61].

### 3.2. Characterisation of Spray Drying-Microencapsulated Powder

The results for *Vaccinium* aqueous extracts (10%) microencapsulated in a maltodextrin (80%) and glucose (20%) matrix are provided in the tables below. As shown in Table 1, the highest yield registered was in the VVIT sample, whereas the lowest was in the VCS sample. The 1% variation between samples can be linked to sample technological recovery. A comparison with similar studies shows that a yield between 79 and 81% is promising, although higher and lower encapsulation efficiencies were also previously reported. Recent research obtained an encapsulation efficiency ranging between 74.4 and 85.22%, which was due to wall material characteristics (18% encapsulating agent) and spray drying conditions [62]. In comparison, a high encapsulation efficiency was obtained during anthocyanin encapsulation from *Hibiscus sabdariffa* L. with a final yield of 99.69% with maltodextrin and 99.87% with gum Arabic [63].

The powder consistency was uniform in all three samples; the colour was white for VMT and VVIT, but pale pink for VCS (data not shown), most likely due to the cyanidin-arabinoside found exclusively in the VCS extract.

### 3.3. Phenolic Profile of Aqueous and Microencapsulated Extracts

The phenolic compounds identified in the aqueous extracts of VCS, VMT, and VVIT using HPLC-DAD-ESI-MS are shown in Table 2. The specificity of each extract resides in the compound name and the subclass it belongs to. As a result, anthocyanins were identified in VCS (particularly cyanidin-arabinoside and cyanidin-glucoside), as previously described in our work [19]. However, hydroxycinnamic acids were primarily discovered in VVIT extract (especially dicaffeoylquinic acid, caffeoylarbutin, feruloylquinic acid I, chlorogenic acid). Flavonol compounds were present in all extracts (9 in VVIT, 8 in VCS and 8 in VMT), as were flavanols (4 in VCS, 4 in VVIT, 3 in VMT).

Prior to microencapsulation, the principal compound was chlorogenic acid, a phenolic acid belonging to the prevalent hydroxycinnamic acids group, which was found in the VMT extract in 104.74 ± 2.23 mg/g, followed by the VCS extract in a quantity of 33.6 ± 0.23 mg/g. The total remaining phenolic content amounted to 10–20% in the microencapsulated extracts, as it can be depicted from Table 2. Significant degradation was observed regarding catechins (gallocatechin, epigallocatechin, procyanidin dimer I, catechin, and epicatechin), which essentially involves structural modifications, oxidation, polymerization, and epimerization producing various by-products with decreased antioxidant potential [64]. Before and after microencapsulation, the highest flavanol content was observed in VCS, although epigallocatechin was also found in the microencapsulated VVIT samples in a quantity of 4.80 ± 0.07 mg/g.

VCS displayed a microencapsulation efficacy of 12.03%, while VMT and VVIT showed a microencapsulation efficacy of 12.26% and 14.72%, respectively. One possible explanation is that phenolic compounds are vulnerable to many factors, such as high temperature and pressure. Hence, phenolic compound degradation is inevitable during the spray-drying microencapsulation process. As the powdered phenolic microcapsules were formed by high-speed homogenization and high-speed spraying phase, external mechanical stress may have caused mechanical damage to phenolic compounds by disrupting phenolic extract interfaces, as well as maltodextrin and casein interfaces [65].

**Table 2:** Phenolic compounds identified in aqueous and microencapsulated extracts of VCS, VMT and VVIT leaves expressed as mg/g.

Peak No.	Retention Time R_t_ (min)	UV λ_max_ (nm)	[M+H]^+^ (*m*/*z*)	Compound	Subclass	Source	VCS		VMT		VVIT	
							Extract	Microcapsules	Extract	Microcapsules	Extract	Microcapsules
1	3.78	279	307, 290	Gallocatechin	Flavanol	VCS, VVIT	1.24 ± 0.01 ^a^	0.21 ± 0.01 ^b^	N.D.	N.D.	0.95 ± 0.01 ^a^	0.16 ± 0.01 ^b^
2	4.69	279	307, 290	Epigallocatechin	Flavanol	VCS, VVIT	1.57 ± 0.01 ^a^	0.25 ± 0.01 ^b^	N.D.	N.D.	32.22 ± 0.32 ^a^	4.80 ± 0.07 ^b^
3	10.36	281, 329	355, 163	3-Caffeoylquinic acid (Neochlorogenic acid)	HA	VCS	5.00 ± 0.09 ^a^	0.45 ± 0.01 ^b^	N.D.	N.D.	N.D.	N.D.
4	11.33	280	579, 291	Procyanidin dimer I	Flavanol	VCS, VMT, VVIT	1.71 ± 0.01 ^a^	0.18 ± 0.01 ^b^	5.55 ± 0.09 ^a^	0.78 ± 0.01 ^b^	5.33 ± 0.08 ^a^	0.88 ± 0.01 ^b^
5	12.21	281, 329	355, 163	5-Caffeoylquinic acid (Chlorogenic acid)	HA	VCS, VMT, VVIT	33.6 ± 0.23 ^a^	3.85 ± 0.02 ^b^	104.74 ± 2.23 ^a^	12.79 ± 0.10 ^b^	6.36 ± 0.04 ^a^	0.64 ± 0.01 ^b^
6	12.76	280	291	(+) Catechin	Flavanol	VCS, VMT, VVIT	1.96 ± 0.02 ^a^	0.18 ± 0.01 ^b^	2.32 ± 0.03 ^a^	0.24 ± 0.01 ^b^	4.76 ± 0.06 ^a^	0.82 ± 0.01 ^b^
7	13.21	280	291	(−) Epicatechin	Flavanol	VCS, VMT	1.87 ± 0.02 ^a^	0.26 ± 0.01 ^b^	1.95 ± 0.02 ^a^	0.19 ± 0.01 ^b^	N.D.	N.D.
8	13.71	282, 329	181, 163	Caffeic acid	HA	VCS, VMT	2.73 ± 0.03 ^a^	0.48 ± 0.01 ^b^	3.06 ± 0.03 ^a^	0.46 ± 0.01 ^b^	N.D.	N.D.
9	14.79	283, 330	369	Feruloylquinic acid I	HA	VCS	2.36 ± 0.02 ^a^	0.30 ± 0.01 ^b^	N.D.	N.D.	N.D.	N.D.
10	15.55	283, 330 263, 355	369 611, 303	Feruloylquinic acid I Quercetin-rutinoside (Rutin)	HA Flavonol	VCS, VVIT	1.76 ± 0.02 ^a^	0.24 ± 0.01 ^b^	N.D.	N.D.	14.77 ± 0.24 ^a^	2.18 ± 0.02 ^b^
11	16.28	263, 355	465, 303	Quercetin-glucoside	Flavonol	VCS, VMT, VVIT	1.86 ± 0.02 ^a^	0.24 ± 0.01 ^b^	14.53 ± 0.21 ^a^	1.76 ± 0.02 ^b^	2.16 ± 0.03 ^a^	0.29 ± 0.01 ^b^
12	16.88	263, 355	625, 303	Quercetin- diglucoside	Flavonol	VCS	0.26 ± 0.01 ^a^	0.03 ± 0.01 ^b^	N.D.	N.D.	N.D.	N.D.
13	17.20	288, 330	435	Caffeoylarbutin	HA	VVIT	N.D.	N.D.	N.D.	N.D.	0.52 ± 0.01 ^a^	0.07 ± 0.01 ^b^
14	17.43	263, 356	493, 303	Quercetin-acetyl-rhamnoside	Flavonol	VCS, VMT, VVIT	0.49 ± 0.01 ^a^	0.10 ± 0.01 ^b^	0.98 ± 0.01 ^a^	0.11 ± 0.01 ^b^	0.98 ± 0.01 ^a^	0.15 ± 0.01 ^b^
15	17.67	262, 355	449, 303	Quercetin-rhamnoside	Flavonol	VMT, VVIT	N.D.	N.D.	1.65 ± 0.02 ^a^	0.19 ± 0.01 ^b^	0.62 ± 0.01 ^a^	0.12 ± 0.01 ^b^
16	18.84	262, 355	435, 303	Quercetin-arabinoside	Flavonol	VMT, VVIT	N.D.	N.D.	0.64 ± 0.01 ^a^	0.08 ± 0.01 ^b^	0.36 ± 0.01 ^a^	0.04 ± 0.01 ^b^
17	19.31	262, 357	596, 303	Quercetin-glucosyl-xyloside	Flavonol	VMT, VVIT	N.D.	N.D.	0.70 ± 0.01 ^a^	0.08 ± 0.01 ^b^	1.96 ± 0.02 ^a^	0.27 ± 0.01 ^b^
18	20.08	282, 329	517, 163	Dicaffeoylquinic acid	HA	VVIT	N.D.	N.D.	N.D.	N.D.	0.57 ± 0.01 ^a^	0.07 ± 0.01 ^b^
19	11.02	210, 517	449, 287	Cyanidin-glucoside	Anthocyanin	VCS, VMT	0.03 ± 0.01	N.D.	0.01 ± 0.01	N.D.	N.D.	N.D.
20	11.78	214, 517	419, 287	Cyanidin-arabinoside	Anthocyanin	VCS	0.03 ± 0.01	N.D.	N.D.	N.D.	N.D.	N.D.
				Total Phenolics			56.47 ± 0.42 ^a^	6.77 ± 0.07 ^b^	136.13 ± 1.23 ^a^	16.68 ± 0.15 ^b^	71.56 ± 0.55 ^a^	10.49 ± 0.12 ^b^

Values (expressed as mean values ± SD, mg/g, *n* = 3) in the same column followed by different letters (a,b) indicate significant differences (*p* < 0.05) between the extract and microencapsulated extract, individual for each type of the three species (one-way analysis of variance (ANOVA); multiple comparison test; Tukey multiple range test (*p* = 0.05); GraphPad Prism Version 8.0.1, Graph Pad Software, Inc., San Diego, CA, USA), UV λmax (nm): ultraviolet absorbance wavelength; [M+H]+ (*m*/*z*): mass spectrometry fragmentation with scanning range of 100–1200 *m*/*z* in the ESI (+) mode, HA—Hydroxycinnamic acid, VMT (*V. myrtillus* L.), VCS (*V. corymbosum* L.), VVIT (*V. vitis-idaea* L.), N.D.—not determined. Table 3 shows the findings on non-encapsulated and microencapsulated VMT aqueous extract before and after in vitro gastrointestinal digestion. Four phenolic compounds were found in both matrices, with chlorogenic acid being the most abundant.

**Table 3:** Effect of salivary, gastric, and intestinal in vitro digestion on the phenolic content of VMT mg/g.

R_t_ (min)	Compound	VMT Microencapsulated				VMT Solution			
		BD	SSF	SGF	SIF	BD	SSF	SGF	SIF
3.85	Quinic acid	N.D.	N.D.	N.D.	1.50 ± 0.01	N.D.	N.D.	N.D.	11.88 ± 0.17
10.39	Protocathecuic acid	N.D.	N.D.	N.D.	1.56 ± 0.01	N.D.	N.D.	N.D.	13.76 ± 0.15
12.21	Chlorogenic acid	12.79 ± 0.10 ^a,b^	12.34 ± 0.09 ^b^	3.65 ± 0.04 ^c^	1.38 ± 0.01 ^d^	104.74 ± 2.23 ^a,b^	96.85 ± 0.87 ^b^	25.54 ± 0.21 ^c^	4.88 ± 0.03 ^d^
16.28	Quercetin-glucoside	1.76 ± 0.02 ^a,b^	1.66 ± 0.02 ^b^	1.16 ± 0.01 ^c^	0.33 ± 0.01 ^d^	14.53 ± 0.21 ^a,b^	14.01 ± 0.10 ^b^	8.10 ± 0.09 ^c^	1.36 ± 0.01 ^d^
	Total Phenolics	14.55 ± 0.12 ^a^	14.00 ± 0.10 ^b^	4.81 ± 0.05 ^c,d^	4.77 ± 0.05 ^d^	119.27 ± 2. 41 ^a^	110.86 ± 2.03 ^b^	33.64 ± 0.32 ^c^	31.88 ± 0.26 ^d^

Values are expressed as mean values ± SD, mg/g, *n* = 3. In the same row, for each type of *V. myrtillus* L. sample (microencapsulated and solution/aqueous extract), values marked with different letters (a–d) indicate a significant difference (*p* < 0.05) between non-digested and after each phase of digestion (One-way analysis of variance (ANOVA); multiple comparison test; Tukey multiple range test (*p* = 0.05); GraphPad Prism Version 8.0.1, Graph Pad Software, Inc., San Diego, CA, USA). VMT (*V. myrtillus* L.), BD—before digestion, SSF—Simulated salivary fluid, SGF—Simulated gastric fluid, SIF—simulated intestinal fluid.

In the case of quercetin-glucoside and chlorogenic acid, the simulated saliva phase exerted practically limited influence on the microcapsules and solution degradation due to the minimal contact time. Considering that the aqueous extract presented a significantly higher initial content of individual phenolics and total phenolic content, phenol values obtained after in vitro digestion are considerably higher than their counterparts (microencapsulated samples). However, the microencapsulated VMT powder presented significantly improved bioaccessibility due to carrier protection compared to the solution. Data is presented below by applying the formula underneath, where initial and final total phenolic values were considered. 

VCS findings before and after in vitro digestion are shown in Table 4 for both microencapsulated and aqueous solution extracts. The same four phenolic compounds in VMT were found in VCS samples, but at lower concentrations. Following exposure to simulated digestion, the total phenolic content in both matrices, microencapsulated and solution (non-encapsulated), reported different values, namely 1.68 ± 0.01 and 13.72 ± 0.09 mg/g, considering the initial phenolic value. On the other hand, chlorogenic acid and quercetin-glucoside were almost entirely consumed or decomposed to other compounds following the digestion process.

Table 5 compares the VVIT species before and after simulated gastrointestinal digestion in microencapsulated and solution extract formulations. Nine phenolic compounds were identified, with the microencapsulated form registering significantly lower total phenolic levels following digestion exposure compared to initial phenolic level, but higher bioaccessibility. However, the majority of the phenolic compounds, particularly five of them (rutin, quercetin-glucoside, caffeoylarbutin, quercetin-acetyl-rhamnoside, and quercetin-rhamnoside), were digested following the intestinal phase, while quercetin-glucosyl-xyloside was nearly wholly degraded. This discovery was made for both types of matrices (microencapsulated and solution). Therefore, the phenolics that are accessible in these extracts were probably already in their free form and the enzymes in the digesting mixture may have interacted with the phenolics, causing oxidation or precipitation. Moreover, variations in pH across the stomach and intestinal phases may induce significant changes in phenolic structure and physicochemical properties [66].

Chlorogenic acids (acyl-quinic acids) are frequently found as secondary metabolites generated by plants, especially in coffee, and possess several health-related prebiotic effects [67,68,69]. These acids comprise a phenolic compound connected by way of an ester bond to a part of quinic acid. This connection through gut microbiota is hydrolysed via esterases, causing the release of quinic or phenolic acids [70]. This is how the production of quinic acid in VCS, VMT, and VVIT can be supported after simulated intestinal fluid digestion. Quinic acid production in the intestine can be attributed to the fact that this compound results from the breakdown of hydroxycinnamic acids, such as caffeoylquinic, feruloyl quinic, chlorogenic acids or other similar more complex compounds. A comparable result has also been reported by El Majdoub et al., 2021, where *Hibiscus sabdariffa* L. (H.s.) extract was subjected to in vitro digestion. This study also revealed that the absorption of polyphenols is affected during gastrointestinal digestion, which diminishes their digestibility. In addition, in the course of the reduction of some compounds (e.g., caffeoylquinicandcoumaroylquinic acids), quinic acid was produced in the duodenal aliquots [71].

As Table 3, Table 4 and Table 5 reveal, total phenolics are obtained for non-encapsulated and microencapsulated extracts of VMT, VCS, and VVIT in three different stages of digestion, namely the salivary, gastric, and intestinal phases, and in their control, namely the extract (microencapsulated extract and solution extract) before digestion, in order to assess and estimate the influence of each stage in the digestion process for both types of samples. However, after gastrointestinal digestion, the concentration of each phenolic component and the total phenolic content decreased in both the microencapsulated and solution formulations. Furthermore, compared to the aqueous extracts, the microencapsulation form revealed a lower concentration of phenolics following each step of digestion in all trials due to higher initial phenolic values in the solution vs. microencapsulated form. However, when translated into bioaccessibility percentages, the microencapsulated form had significantly higher values than their counterparts.

For instance, reactions of deconjugation and reconjugation can metabolize phenolic compounds. Phenolics are hydrolysed to their free aglycones, then conjugated by methylation, sulfation, or glucuronidation. The majority of flavonoids found in plants are glycosylated, which means they are related to sugars like glucose, glucorhamnose, galactose, arabinose, and rhamnose. The binding of these sugars of the flavonoids is not hydrolysed by pancreatic enzymes during digestion, implying that the released flavonoids stay glycosylated even after the in vitro digestion process, making them more stable in the medium circumstances. On the other hand, two endoglucosidases found in the human small intestine, enzymes not seen in an in vitro digesting process, can cleave this link. Moreover, flavonols might bind to proteins or fibres in the matrix through hydrogen bonding, covalent bonding or hydrophobic interactions. During exposure to gastric and intestinal conditions (pH and enzymes), the solubility of these compounds may change, impacting the bioaccessibility. Regarding the phenolic acids, the changes during the in vitro digestion might be explained by oxidation or degradation, as well as their distribution in the vegetal tissue (the distinct structure formed by pectin, cellulose and hemicellulose in the vegetable tissue). The metabolic pathway is similar to drug metabolism, but there are differences between phenolic compounds and drugs. Drugs are usually administered in milligrams in one concentrated dose, while phenolics are consumed in lower quantities. The drugs rapidly saturate the metabolic pathways, whereas the phenolics cannot [72]. 

### 3.4. Bioaccessibility of Phenolic Compounds

Bioaccessibility can be comprehended as the quantity of a compound discharged within the gastrointestinal tract and accessible for absorption. The bioaccessibility of these bioactive compounds is important and proficient in modulating certain metabolic processes, thus achieving health improvement [73]. In terms of bioaccessibility (Table 6), only four compounds were identified in VMT and VCS digested samples. During quantification and salivary-gastrointestinal digestion, reference was made to quinic acid, protocathecuic acid, 5-caffeoylquinic acid (chlorogenic acid), and quercetin-glucoside. Therefore, bioaccessibility was calculated according to the following formula:

$$Bioacessibility\;\left(\%\right)=\frac{phenolic\;content\;released\;after\;the\;gastrointestinal\;digestion*}{phenolic\;content\;in\;chemical\;extract}\times 100$$

* Phenolic content was measured in the digested supernatant after centrifugation.

**Table 6:** Bioaccessibility of VMT, VCS and VVIT aqueous and microencapsulated extracts.

Extract	Bioaccessibility (%)
VMT	26.72%
VMT Microencapsulated	32.78%
VCS	38.65%
VCS Microencapsulated	41.07%
VVIT	34.04%
VVIT Microencapsulated	45.43%

Abbreviations used: VMT (*V. myrtillus* L.), VCS (*V. corymbosum* L.), and VVIT (*V. vitis-idaea* L.)—plant species.

The microencapsulated formulation of VVIT and VCS extracts had the highest bio accessibility values, 45.43%, and 41.07%, respectively, whereas VCS had a high value for non-encapsulated samples, 38.65%. Similar studies also report polyphenol bioaccessibility is the highest around 40%, for orange juice between 11–36%, for broccoli 6% and between 19–39 for different bean types [74].

As illustrated previously, the quantity of phenolic compounds through in vitro digestion decreases or increases, based on the digestive stage. For example, chlorogenic acid is mainly bioaccessible in the stomach phase due to its absorption through the gastric mucosa [75]. Quinic acid, an esterified compound, is absorbed much harder in the gastric and intestinal phase, resisting against pH alterations and enzymes [76].

## 4. Conclusions

After the in vitro digestion model, aqueous extracts of *Vaccinium* spp. (bilberry, lingonberry, and blueberry) were successfully microencapsulated and assessed in terms of the bioaccessibility of their phenolic components. Sample viscosity was evaluated preceding microencapsulation, resulting in a shear thickening behaviour under a shear stress of 50 s^−1^ and above a Newtonian behaviour. The final viscosity of the samples ranged between 1.024 and 1.049 mPa·s. For these leaf extracts, the spray drying-microencapsulated powder was effectively improved, with the highest yield recorded in the VVIT sample and the lowest in the VCS. The 1% variation between samples, on the other hand, can be attributed to sample technical recovery.

Following gastrointestinal digestion, the content of each phenolic component and the total phenolic content decreased significantly in both microencapsulated and aqueous formulations. Compared to the water solution, the microencapsulation version of extracts had a greater bioaccessibility of phenolics after digestion. The highest bioaccessibility values were registered for the microencapsulated form of VVIT and VCS extracts (45.43% and 41.07%), while VCS showed a high value for non-encapsulated samples (38.65%).

The microencapsulation of *Vaccinium* spp. (Bilberry, lingonberry, and blueberry) aqueous extracts reveals the tremendous functional use of the leaves, with considerable implications in the field. Further in vivo research is needed to evaluate the bioavailability of the chemicals found in the examined leaves and their beneficial impact on human health.

## Figures and Tables

**Table 1:** Results obtained for plant-based powders.

Sample	Initial Quantity	Final Quantity	Yield
	(Dry Weight) g	(Powder) g	(Final Quantity/Initial Quantity × 100)
VMT	120 mL for feeding consisting of 44 ± 1.2 g dry weight/volume carrier	35.21 ± 0.5 g powder	35.21/44 × 100 yield approx. 80%
VCS	135 mL for feeding consisting of 48.5 ± 0.9 g dry weight/volume carrier	38.37 ± 1.5 g powder	38.37/48.5 × 100 yield approx. 79%
VVIT	105 mL for feeding consisting of 38.5 ± 1.4 g dry weight/volume carrier	31.12 ± 2.3 g powder	31.12/38.5 × 100 yield approx. 81%

The microencapsulation yield results for Vaccinium aqueous extracts (10%) microencapsulated in maltodextrin (80%) and glucose (20%) matrix are provided in the present table. Plant leaf species used: VMT (*V. myrtillus* L.), VCS (*V. corymbosum* L.), and VVIT (*V. vitis-idaea* L.).

**Table 4:** Effect of salivary, gastric and intestinal in vitro digestion model on the phenolic content of VCS mg/g.

R_t_ (min)	Compound	VCS Microencapsulated				VCS Solution			
		BD	SSF	SGF	SIF	BF	SSF	SGF	SIF
3.85	Quinic acid	N.D.	N.D.	N.D.	0.84 ± 0.01	N.D.	N.D.	N.D.	6.56 ± 0.05
10.39	Protocathecuic acid	N.D.	N.D.	N.D.	0.72 ± 0.01	N.D.	N.D.	N.D.	6.44 ± 0.05
12.28	5-Caffeoylquinic acid (Chlorogenic acid)	3.85 ± 0.02 ^a,b^	3.55 ± 0.02 ^b^	1.85 ± 0.01 ^c^	0.12 ± 0.01 ^d^	33.63 ± 0.23 ^a^	29.85 ± 0.19 ^b^	16.38 ± 0.11 ^c^	0.72 ± 0.01 ^d^
16.28	Quercetin-glucoside	0.24 ± 0.01 ^a,b^	0.20 ± 0.01 ^b^	0.14 ± 0.01 ^c^	0.00	1.86 ± 0.02 ^a,b^	1.78 ± 0.02 ^b^	0.86 ± 0.01 ^c^	N.D.
	Total Phenolics	4.09 ± 0.03 ^a,b^	3.75 ± 0.02 ^b^	1.99 ± 0.01 ^c,d^	1.68 ± 0.01 ^d^	35.49 ± 0.25 ^a^	31.63 ± 0.20 ^b^	17.24 ± 0.10 ^c^	13.72 ± 0.09 ^d^

Values are expressed as mean values ± SD, mg/g, *n* = 3. In the same row, for each type of *V. corymbosum* L. sample (microencapsulated and solution/aqueous extract), values marked with different letters (a–d) indicate a significant difference (*p* < 0.05) between non-digested and after each phase of digestion (One-way analysis of variance (ANOVA); multiple comparison test; Tukey multiple range test (*p* = 0.05); GraphPad Prism Version 8.0.1, Graph Pad Software, Inc., San Diego, CA, USA), VCS (*V. corymbosum* L.), BD—before digestion, SSF—Simulated salivary fluid, SGF—Simulated gastric fluid, SIF—simulated intestinal fluid.

**Table 5:** Effect of salivary, gastric and intestinal in vitro digestion model on the phenolic content of VVIT mg/g.

Rt (min)	Compound	VVIT Microencapsulated				VVIT Solution			
		BD	SSF	SGF	SIF	BD	SSF	SGF	SIF
3.85	Quinic acid	N.D.	N.D.	N.D.	1.02 ± 0.01	N.D.	N.D.	N.D.	6.76 ± 0.05
4.59	Epigallocatechin	4.80 ± 0.07 ^a^	4.13 ± 0.05 ^b^	2.79 ± 0.01 ^c^	1.54 ± 0.01 ^d^	32.22 ± 0.32 ^a^	26.60 ± 0.21 ^b^	11.10 ± 0.08 ^c^	5.72 ± 0.04 ^d^
10.39	Protocatechuic acid	N.D.	N.D.	N.D.	0.92 ± 0.01	N.D.	N.D.	N.D.	4.88 ± 0.05
15.45	Feruloylquinic acid I Quercetin-rutinoside (Rutin)	2.18 ± 0.02 ^a,b^	2.00 ± 0.02 ^b^	0.39 ± 0.01 ^c^	N.D.	14.77 ± 0.24 ^a^	11.30 ± 0.09 ^b^	4.98 ± 0.03 ^c^	N.D.
16.28	Quercetin-glucoside	0.29 ± 0.01 ^a,b^	0.20 ± 0.01 ^b^	0.13 ± 0.01 ^c^	N.D.	2.16 ± 0.03 ^a^	1.94 ± 0.01 ^b^	0.96 ± 0.01 ^c^	N.D.
17.20	Caffeoylarbutin	0.07 ± 0.01 ^a,b,c^	0.06 ± 0.01 ^b,c^	0.04 ± 0.01 ^c^	N.D.	0.52 ± 0.01 ^a^	0.42 ± 0.01 ^b,c^	0.40 ± 0.01 ^c^	N.D.
17.42	Quercetin-acetyl-rhamnoside	0.15 ± 0.01 ^a,b^	0.14 ± 0.01 ^b^	0.06 ± 0.01 ^c^	N.D.	0.98 ± 0.01 ^a^	0.73 ± 0.01 ^b^	0.62 ± 0.01 ^c^	N.D.
17.65	Quercetin-rhamnoside	0.12 ± 0.01 ^a,b^	0.10 ± 0.01 ^b,c^	0.08 ± 0.01 ^c^	N.D.	0.62 ± 0.01 ^a^	0.57 ± 0.01 ^a,b^	0.50 ± 0.01 ^b,c^	N.D.
19.30	Quercetin-glucosyl-xyloside	0.27 ± 0.01 ^a,b^	0.26 ± 0.01 ^b^	0.14 ± 0.01 ^c^	0.10 ± 0.01 ^d^	1.96 ± 0.02 ^a,b^	1.94 ± 0.02 ^b^	1.42 ± 0.01 ^c^	0.76 ± 0.01 ^d^
	Total Phenolics	7.88 ± 0.14 ^a^	6.89 ± 0.10 ^b^	3.63 ± 0.02 ^c,d^	3.58 ± 0.01 ^d^	53.23 ± 0.64 ^a^	43.50 ± 0.32 ^b^	19.98 ± 0.17 ^c,d^	18.12 ± 0.10 ^d^

Values are expressed as mean values ± SD, mg/g, *n* = 3. In the same row, for each type of *V. vitis-idaea* L. sample (microencapsulated and solution/aqueous extract), values marked with different letters (a–d) indicate a significant difference (*p* < 0.05) between non-digested and after each phase of digestion (One-way analysis of variance (ANOVA); multiple comparison test; Tukey multiple range test (*p* = 0.05); GraphPad Prism Version 8.0.1, Graph Pad Software, Inc., San Diego, CA, USA), VVIT (*V. vitis-idaea* L.), BD—before digestion, SSF—Simulated salivary fluid, SGF—Simulated gastric fluid, SIF—simulated intestinal fluid.

## Data Availability

Data is contained within the article.
